# Multi-omics integration uncovers key transcriptional regulators in triple-negative breast cancer spatial heterogeneity

**DOI:** 10.3389/fgene.2025.1614254

**Published:** 2025-09-03

**Authors:** Ning Zhang, Ye Zhang, Rui-Fei Yang, Min Tan, Hai-Qing Chu, Abdur Rehman, Lu-Yu Yang, Ya-Yu Li, Fahad M. Alshabrmi, Xin Zhou, Feili Xu, Shou-Ping Gong, Hui-Ling Cao

**Affiliations:** ^1^ Xi’an Key Laboratory of Basic and Translation of Cardiovascular Metabolic Disease, Xi’an Key Laboratory of Autoimmune Rheumatic Disease, College of Pharmacy, Xi’an Medical University, Xi’an, China; ^2^ College of Life Sciences, Northwest A&F University, Yangling, Shaanxi, China; ^3^ College of Health Management, Shangluo University, Shangluo, Shaanxi, China; ^4^ Xi’an Siyuan University, Xi’an, China; ^5^ Department of Medical Laboratories, College of Applied Medical Sciences, Qassim University, Buraydah, Saudi Arabia

**Keywords:** triple-negative breast cancer, spatial transcriptomics, microenvironment, tumor spread, cell communication

## Abstract

**Background:**

Triple-negative breast cancer (TNBC) is an aggressive subtype of breast cancer characterized by a lack of hormone receptors, making it challenging to treat.

**Methods:**

We generated a comprehensive spatial cell atlas of TNBC using a multi-omics integration approach that combined single-cell RNA sequencing (scRNA-seq) with spatial transcriptomics. This integration allowed us to characterize the spatial microenvironment and map the cell-type-specific distributions in TNBC tissues.

**Results:**

Our analysis revealed significant heterogeneity in cell types and spatial distribution, with normal regions enriched in insulin resistance functions, whereas cancerous regions displayed diverse cell populations, including immune cells, cancer-associated fibroblasts (CAFs), and mesenchymal cells. By constructing transcription factor (TF) regulatory networks, we identified TFF3, RARG, GRHL1, RORC, and KLF5 as critical regulators of epithelial cells, whereas EMX2, TWIST1, TWIST2, NFATC4, and HOXC6 were found to play essential roles in mesenchymal cells. Immunohistochemical validation supported the involvement of these TFs in TNBC. Further analysis of receptor-ligand interactions highlighted the roles of KNG1_BDKRB2 and NRG1_ERBB4 signaling in promoting tumor aggression, suggesting potential therapeutic targets. GO enrichment analysis revealed overlapping pathways between epithelial and mesenchymal cells, focusing on migration, signaling, and development, indicating that the shared regulatory mechanisms contribute to cancer progression.

**Conclusion:**

Our findings provide new insights into the TNBC microenvironment, emphasizing the complex spatial interactions between different cell types and highlighting key regulatory pathways that could be targeted for future therapeutic interventions. This spatial cell atlas lays the foundation for further exploration of tumor microenvironment dynamics and precision oncology approaches.

## 1 Introduction

Breast cancer is the most prevalent cancer diagnosed among women worldwide, affecting approximately 1.7 million women annually and resulting in approximately 500,000 deaths worldwide. (Bray et al., 2018; Ferlay et al., 2015; Siegel et al., 2018; Rodrigue et al., 2019). Triple-negative breast cancer (TNBC) is considered one of the most severe subtypes, accounting for 10%–20% of breast cancer diagnoses, and is defined by the lack of expression of the estrogen receptor (ER), progesterone receptor (PR), and HER2 (Zhao et al., 2021; Abu-Jamous et al., 2017). Owing to its high aggressiveness and unfavorable prognosis, TNBC is often linked to hypoxic conditions and significant intratumor heterogeneity (Abu-Jamous et al., 2017; Saatci et al., 2020). This heterogeneity poses a major challenge to effective treatment, leading to limited therapeutic options compared with non-TNBC subtypes.

The etiology of breast cancer is complex and involves a combination of genetic predispositions, environmental factors, and high-risk conditions such as obesity, hormonal imbalances, and early or late menarche. The tumor microenvironment (TME) is vital for driving tumor growth and therapy resistance. For instance, TNBC cells secrete cytokines such as granulocyte-macrophage colony-stimulating factor (GM-CSF), which stimulates the growth of hematopoietic and myeloid cells, thereby contributing to the reactive stroma observed in TNBC (Hanahan and Weinberg, 2011).

Advances in scRNA-seq have facilitated the detailed analysis of intra-tumor gene expression variability (Patel et al., 2014; Kim Et Al., 2015; Grun et al., 2015; Junker and Van Oudenaarden, 2015). However, scRNA-seq lacks spatial context, making it difficult to understand the interaction of tumor cells with their microenvironment (Satija et al., 2015; Achim et al., 2015). Earlier *in situ* sequencing approaches were restricted to analyzing a limited set of genes (Ke et al., 2013; Lee et al., 2014), and the spatial transcriptomic profile of TNBC was scarcely studied prior to the development of Spatial Transcriptomics (ST).

In this study, we used a multifocal TNBC model to investigate the spatial and transcriptomic features of TNBC. By combining scRNA-seq and ST methods, we aimed to capture the transcriptomic heterogeneity of TNBC cells and their microenvironment, thereby offering deeper insights into the molecular mechanisms underlying tumor growth and cell-to-cell communication. This integrative approach allows us to uncover critical regulatory networks and identify new therapeutic targets within the spatial context of TNBC tissue (Stahl et al., 2016).

## 2 Methods

### 2.1 Data collection and analysis

The annotated cell types and breast cancer dataset were sourced from the Gene Expression Omnibus (GEO) database GSE176078 (Wu et al., 2021). These data were divided into two parts: (1) scRNA-seq was performed on tumor samples from 10 patients with triple-negative breast cancer (TNBC) aged between 39 and 73 years. All patients were diagnosed with grade III advanced-stage invasive carcinoma, predominantly classified as either invasive ductal carcinoma (IDC) or metaplastic breast carcinoma (MBC). (2) ST data of 1 TNRC (patient CID44971). A summary of the patients’ clinical and pathological features is provided in Supplementary Table S1.

The KM-plotter tool which includes clinical cohorts from GEO, European Genome-phenome Archive (EGA), and The Cancer Genome Atlas (TCGA) was used to evaluate the correlation between gene expression and survival rates in 335 TNBC patients (https://kmplot.com/analysis/) (Gyorffy, 2021).

### 2.2 scRNA-seq data processing and cell type annotation

Gene-barcode counts matrices were analyzed using the Seurat R package (version 4.0.4) (https://www.satijalab.org/seurat). Cells with fewer than 200 detected genes or with more than 20% mitochondrial gene content were excluded. The samples were then merged into a single Seurat object. The merged Seurat object was normalized with regression on UMI count and mitochondrial content and mitochondrial gene percentage. The characteristics of the dataset were based on a list of 1,500 most variable genes (FindVariableFeatures). t-SNE was applied to the top 30 PCs using Seurat’s RunTSNE.

**FIGURE 1:**
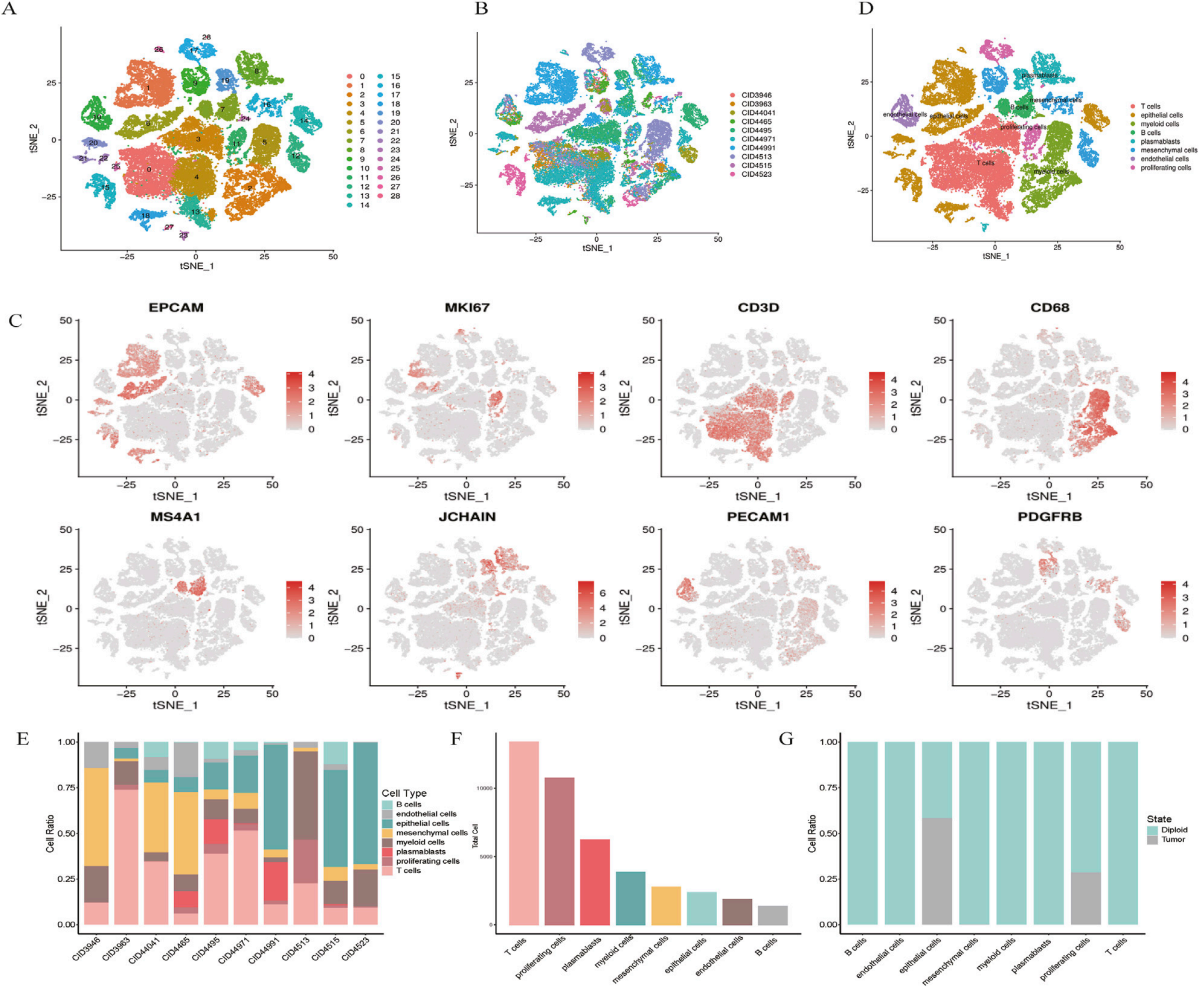
scRNA-seq map of breast cancer. **(A)** t-SNE plot showing the clustering of scRNA-seq expression profiles. **(B)** t-SNE representation of all 48,164 cells, annotated by patient. **(C)** Marker gene expression visualized for each cluster on t-SNE. **(D)** t-SNE clustering by cell types, including epithelial, myeloid, T cells, B cells, plasmablasts, mesenchymal, endothelial, and proliferating cells. **(E,F)** Bar plots illustrating cell type distribution by patient, tumor versus normal origin, and total cell count. **(G)** Normal and tumor cell distribution patterns.

Cell types were defined using a combination of unsupervised clustering and differential expression, whereby we compared the t the most differentially expressed genes were compared to known cell types. The cells were classified into broad categories, and cellular subtypes were further delineated by isolating subsets (through *in silico* “gating”) of broadly defined cell types, followed by re-analysis using the same procedure. A total of 29 clusters were identified for wide cell type annotation (Figure 1A). For optimal clustering, resolutions of 0.4 were used for the data shown in the present study. The optimal clustering resolution in Seurat was determined by clustering integrated single-cell expression data at 10 different resolutions from 0.1 to 1.0 using the “resolution” parameter in the FindClusters () function. The clusters of interest were filtered and compared for differential gene expression using the Wilcoxon rank-sum test (FindAllMarkers function; only. pos = TRUE, min. pct = 0.25, logfc. threshold = 0.5, p-value cut-off = 0.05) to discover marker genes or upregulated genes. Markers for epithelial (EPCAM), proliferative (MKI67), T cells (CD3D), myeloid (CD68), B cells (MS4A1), plasmablasts (JCHAIN), endothelial (PECAM1), and mesenchymal cells (PDGFRB) was log-normalized.

### 2.3 Tumor cells with their normal counterparts analysis alone

CNV analysis in scRNA-seq is a powerful approach to distinguish cancer cells from normal cells by identifying significant chromosomal variations, such as gains or losses of large DNA segments, through gene expression patterns along chromosomes. This method estimates DNA copy numbers in specific genomic regions based on RNA expression levels, enabling the detection of cells with substantial CNV alterations, like cancer cells, compared to diploid cells. In this study, CopyKAT (Gao et al., 2021) was employed to differentiate tumor cells from normal cells using high-throughput scRNA-seq data. Tumor cells exhibit genome-wide aneuploidy, whereas stromal and immune cells generally show 2N or near-diploid profiles.

### 2.4 Integration with spatial transcriptome and single-cell data

To explain the spatial microenvironment of TNBCs, we first need to obtain a spatial gene expression map at single-cell resolution. We integrated the ST of TNBCs (CID44971) with the single-cell transcriptome for annotation to create a more accurate annotation file between samples. Next, the filtered single-cell and spatial transcriptomic data (CID44971) ensured reliability by excluding aberrant cells.

scRNA-seq data from adjacent sections of human breast cancer (CID44971) annotated using a deep learning-based deconvolution algorithm based on Tangram software to validate the cell type distribution pattern of breast cancer pathology sections. (Biancalani et al., 2021). Briefly, the “rank_genes_groups” function in Scanpy was used to identify marker genes in single-cell clusters (Wolf et al., 2018). The top 20 DEGs ranked by fold change were selected as input marker genes. To map scRNA-seq-defined cell types onto the ST data, we employed Tangram, a probabilistic deep learning-based deconvolution framework that aligns single-cell profiles to spatial transcriptomic spots. Tangram leverages both marker genes and global transcriptomic features to achieve robust and biologically consistent mapping, even in the presence of microenvironmental heterogeneity or patient-specific variation. Following quality control to remove aberrant cells or spots, a total of 1,162 high-quality spots from the ST dataset were retained for downstream spatial mapping. Tangram then projected the scRNA-seq-inferred cell types onto these spatial spots, and the resulting normalized spatial cell-type probability distributions were visualized across the tissue section.

### 2.5 Spatial cell-cell interaction analysis

The Python toolbox stLearn was used to analyze spatial cell communication within ST data from TNBCs, as described by Pham (Pham et al., 2020). This tool is recognized for its ability to normalize gene expression by leveraging morphological similarities in adjacent regions, effectively reducing the “dropout” noise associated with RNA-seq technology limitations. Genes detected in fewer than three locations were excluded from the analysis of TNBC ST-seq data. The filtered gene counts were normalized to counts per million (CPM), log-transformed, and scaled. For ligand-receptor (L-R) predictions, we applied CellPhoneDB (v3.0.0) with default parameters and the curated database version from Efremova et al. (2020). Additionally, NicheNet was employed for L-R predictions on 10× Genomics Visium ST data, following a similar approach to snRNA-seq for cell-cell communication analysis through L-R pairings. Finally, the gene count matrix was normalized using stSME, which incorporates tissue morphology into the process.

### 2.6 Inference of regulons and their activity

A multi-species-adapted version of the SCENIC pipeline was utilized to reconstruct gen GRNs from scRNA-seq datasets (Aibar et al., 2017; Davie et al., 2018; Han et al., 2018). This approach includes three main steps: (1) determining co-expression modules between transcription factors (TFs) and their potential target genes; (2) identifying direct target genes for each module by detecting motifs enriched for specific TFs; and (3) calculating the Regulon Activity Score (RAS) in individual cells using the area under the recovery curve.

SCENIC software systematically identified key regulators associated with epithelial cell function and mesenchymal cell identity. For each regulon, we assessed activity patterns specific to the two major cell types and derived a Regulon Specificity Score (RSS) using Jensen-Shannon divergence. Regulons with the highest RSS values were prioritized for functional analysis.

In this study, “species” refers to the replacement of default mouse-based settings in SCENIC with human-specific transcription factor annotations and motif databases, ensuring biological relevance and improving GRN inference accuracy in human TNBC samples. To determine the cell-type specificity of a regulon, an entropy-based method previously applied for gene expression analysis was utilized (Cabili et al., 2011). Regulators with the highest cell type-specific scores were identified as the most significant for each cell type.

To identify regulon modules, we applied the Connection Specificity Index (CSI), a context-aware metric for detecting associations (Bass et al., 2013). Using hierarchical clustering based on Euclidean distance, distinct regulon modules were delineated from the CSI matrix. A threshold of CSI >0.7 was adopted to establish a regulon association network. Similarly, submodules within M7 were identified using this approach. For each regulon module, cell type-specific activity scores were calculated as the average activity of its members across all cells of a given type. The top-ranked cell types were determined for each module.

### 2.7 Cell culture and real-time PCR

MCF7 cells were maintained in MEM NEAA (Procell, China) medium supplemented with 10% fetal bovine serum (Sijiqing, Hangzhou, China) and penicillin/streptomycin (Solarbio Science and Technology Co. Ltd., Beijing, China). The cells were cultured in a humidified atmosphere containing 95% air and 5% CO_2_ at 37 °C.

MCF7 cells were treated with or without oxymatrine (12.5 μg/mL) for 72 h (24 h pretreatment, followed by another 48 h incubation with/without stimulation). Total RNA was obtained using a commercial total RNA purification kit (Axygen) and subsequently subjected to reverse transcription using a cDNA synthesis kit (TaKaRa) following the manufacturer’s instructions. Primer sequences used for qPCR were: EMX2 (forward:′TCATCCACCGCTACCGATATCTG′; Reverse: ‘TGTTGCGAATCTGAGCCTTCTTC’),TFF3(forward: ‘CAT​GCT​GGG​GCT​GGT​CCT​G′; Reverse: ‘GGCACGGCACACTGGTTTG′)RARG (forward:′CGCCGAAGCATCCAGAAGAAC’; Reverse:′GCGATTCCTGGTCACCTTGTTG′),and GAPDH (forward: 5′- GGA​GTC​CAC​TGG​CGT​CTT-3′; reverse: 5′- AGG​CTG​TTG​TCA​TAC​TTC​TCA​T-3′). Real-time PCR analysis was performed using SYBR Green PCR Premix Ex Taq II reagents (TaKaRa) on a real-time PCR system (Tianlong, Gentier 96E, CHINA). GAPDH served as the housekeeping gene. qPCR results were analyzed using the comparative CT method.

## 3 Results

### 3.1 Multimodal profiling of breast cancer

Single-cell RNA sequencing (scRNA-seq) was conducted on tumor samples from 10 TNBC patients. A total of 50,009 cells were sequenced, with 48,164 cells passing quality control filters and subsequently analyzed using Seurat. Unsupervised clustering revealed distinct populations of tumor cells, normal epithelial cells, stromal cells, and immune subtypes (Figures 1A,B). Notably, clustering was predominantly influenced by cell type rather than by batch variation.

Marker gene analysis confirmed the identities of various cell types, including epithelial cells (EPCAM), proliferative cells (MKI67), T cells (CD3D), myeloid cells (CD68), B cells (MS4A1), plasmablasts (JCHAIN), endothelial cells (PECAM1), and mesenchymal cells (PDGFRB) (Figure 1C). The distribution of these cell types across patients and tumor samples demonstrated consistent diversity (Figures 1E,F).

Using the Infer CNV algorithm, we predicted copy number variations (CNVs) to assess the genomic heterogeneity between tumor and normal cells. We identified 529 malignant cells with significant chromosomal alterations, distinguishing them from non-malignant cells. Interestingly, proliferative and endothelial cells did not exhibit significant CNV, suggesting that these cells may retain a more stable genome (Figure 1G). Taken together, these results provide a comprehensive cellular atlas of TNBC, highlighting the heterogeneity of both the tumor and stromal compartments.

### 3.2 Breast cancer spatial cell communication

**FIGURE 2:**
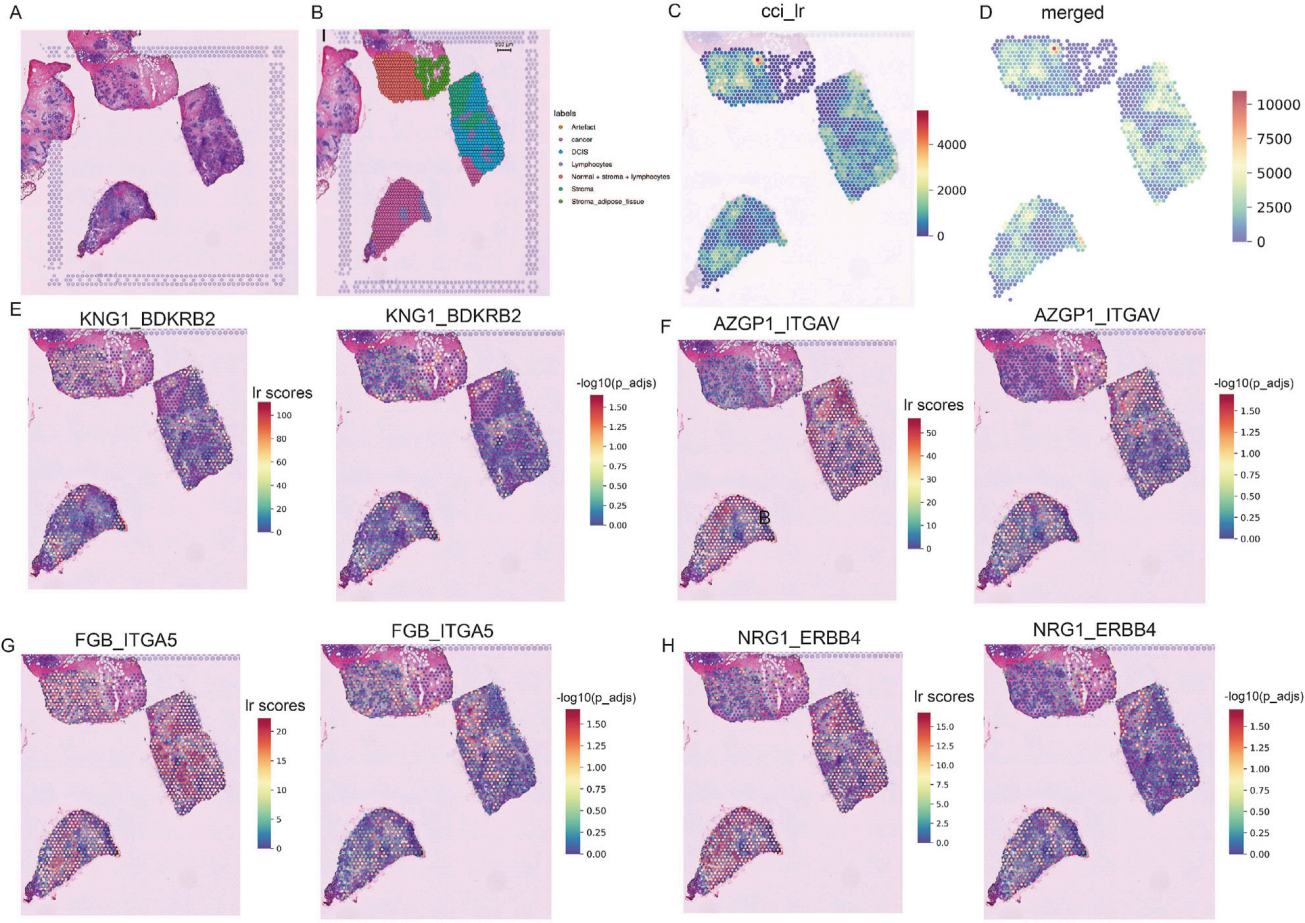
Spatially resolved transcriptomics to analyze TNBC heterogeneity. **(A)** Pathological sections of TNBC. **(B)** Morphological annotation of tissue regions in sample TNBC CID44971, including categories such as cancer, DCIS, lymphocytes, stroma, and adipose tissue. **(C)** Ligand-receptor co-expression in neighboring spots. **(D)** Merged cell-cell interaction (CCI) scores between spots. **(E–H)** TNBC-specific spatial receptor-ligand communication scores with corresponding -log (p_adj) values.

We conducted spatial transcriptome analysis on the TNBC sample CID44971 to explore the spatial organization and cellular interactions within the TME. Pathological sections were annotated to distinguish between cancerous regions, ductal carcinoma *in situ* (DCIS), lymphocytes, normal stroma, and adipose tissue (Figures 2A,B).

Using the stLearn tool, we examined L-R co-expression within spatial transcriptomic spots (Pham et al., 2020). We identified more than 1000 L-R combinations from the CellPhoneDB database (Browa et al., 2020), revealing significant differences in cellular communication between cancerous and stromal regions (Figures 2C,D). In particular, communication was more active in the normal stroma lymphocyte region, whereas cancerous regions displayed spatial heterogeneity, with areas of both dense and sparse cellular communication (Figure 2C).

Further analysis identified several receptor-ligand pairs with elevated activity in the tumor region. Among these, KNG1_BDKRB2 stands out because of its strong association with cancer prognosis and high expression within the tumor area (Figure 2E) (Cui et al., 2020). This suggests that KNG1_BDKRB2 may be involved in tumor progression. NRG1_ERBB4 activates the YAP transcriptional coactivator, promoting cell growth and migration via the Hippo pathway and contributing to tumor aggressiveness (Figure 2H) (Haskins et al., 2014). FGB_ITGA5 and AZGP1_ITGAV: These pairs may be potential targets for inhibiting cancer spread or progression (Figures 2F,G). These findings underscore the complex spatial organization of TNBC and highlight key receptor-ligand interactions that may serve as therapeutic targets.

### 3.3 Prediction of different cell types in spatial spots of breast cancer tissue sections

**FIGURE 3:**
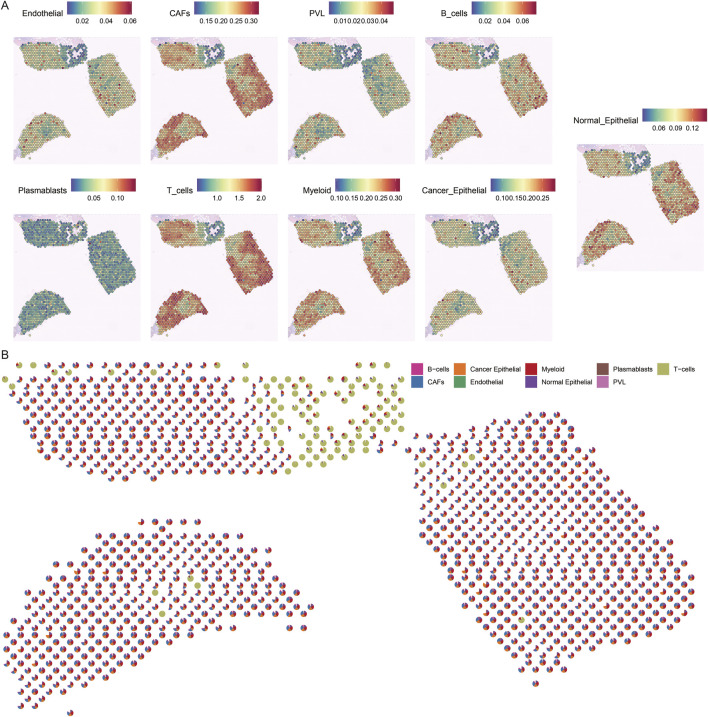
Spatial mapping of cell types in breast cancer tissues. **(A)** Spatial plot of predicted cell type distribution across captured spots. **(B)** Spatial plot of predicted cell type compositions within captured spots.

To gain insight into the cellular composition of TNBC, we first annotated the single-cell transcriptome data of TNBC samples (CID44971). After quality control, we used the Tangram tool to integrate and deconvolute single-cell and spatial transcriptome data, identifying major cell types, including endothelial cells, CAFs, perivascular-associated fibroblasts (PVL), B cells, T cells, myeloid cells, and both cancerous and normal epithelial cells (Figure 3A).

The spatial distribution of these cell types revealed distinct patterns. For instance, cancer cells were found to coexist with fibroblasts, albeit in varying proportions across different regions. In contrast, normal epithelial cells were localized to specific regions of the tissue slices. Notably, immune cells, such as B cells and T cells, were significantly more abundant in normal regions, whereas cancerous regions displayed a more diverse and complex cell composition (Figure 3B).

These findings highlight the spatial heterogeneity of TNBC, where immune cells dominate the normal tissue environment, while the TME is enriched with a more diverse array of cell types, reflecting the complexity of cancer progression.

### 3.4 Spatial mapping of breast cancer heterogeneity

**FIGURE 4:**
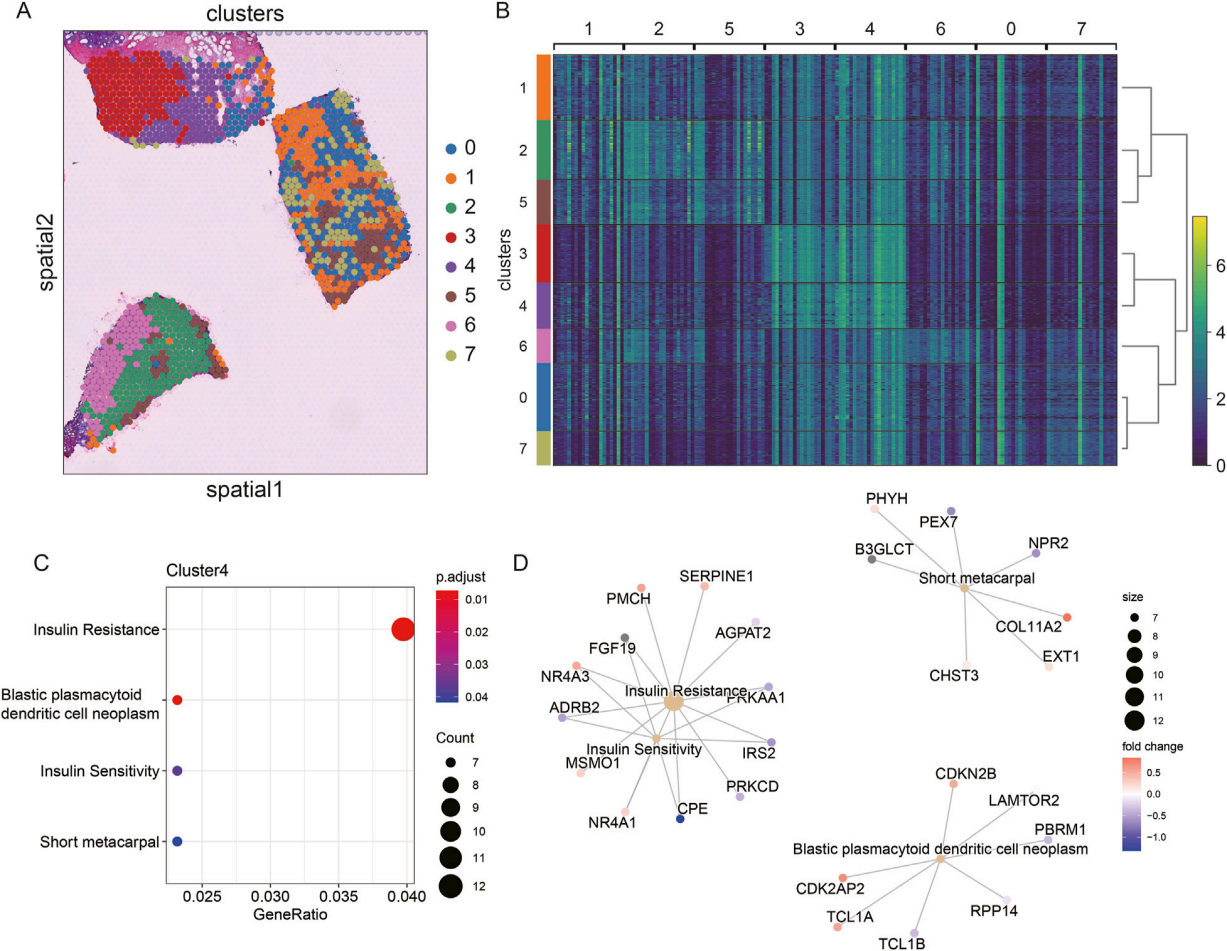
Spatial transcriptome slicing of TNBCs (CID44971) clustering and GO enrichment. **(A)** Clustering of Spatial transcriptomes TNBCs (CID44971). **(B)** Heat map of spatial transcriptome clustering of TNBCs (CID44971). **(C)** Slices normal region (cluster4) GO functionally enriched. **(D)** Slices normal region (cluster4) GO functionally enriched for participating genes.

To explore the spatial heterogeneity of TNBC, we performed clustering analysis on the spatial transcriptome data of TNBC (CID44971). The analysis revealed eight distinct clusters, with cancer regions primarily corresponding to clusters 2 and 6, while ductal carcinoma *in situ* (DCIS), lymphocytes, and stromal regions corresponded to clusters 0, 1, 5, and 7 (Figures 4A,B).

To better understand the resistance of normal tissue to tumor progression, we conducted GO enrichment analysis of the marker genes from cluster 4, which was primarily associated with normal tissue. The enriched pathways included insulin resistance and blast plasmacytoid dendritic cell neoplasms (Figure 4C). This suggests that the normal regions are primarily enriched in metabolic processes, such as insulin resistance, which could play a protective role against tumor invasion.

Further regulatory network analysis revealed that insulin resistance and sensitivity pathways were modulated through key genes, including IRS2, CPE, ADRB2, NR4A3, and FGF19 (Figure 4D). These interactions highlight a potential metabolic regulatory mechanism that distinguishes normal tissue from cancerous regions, suggesting that insulin resistance may serve as a key barrier to tumor spread.

### 3.5 Comparative analysis of essential regulators for cell identity maintenance

**FIGURE 5:**
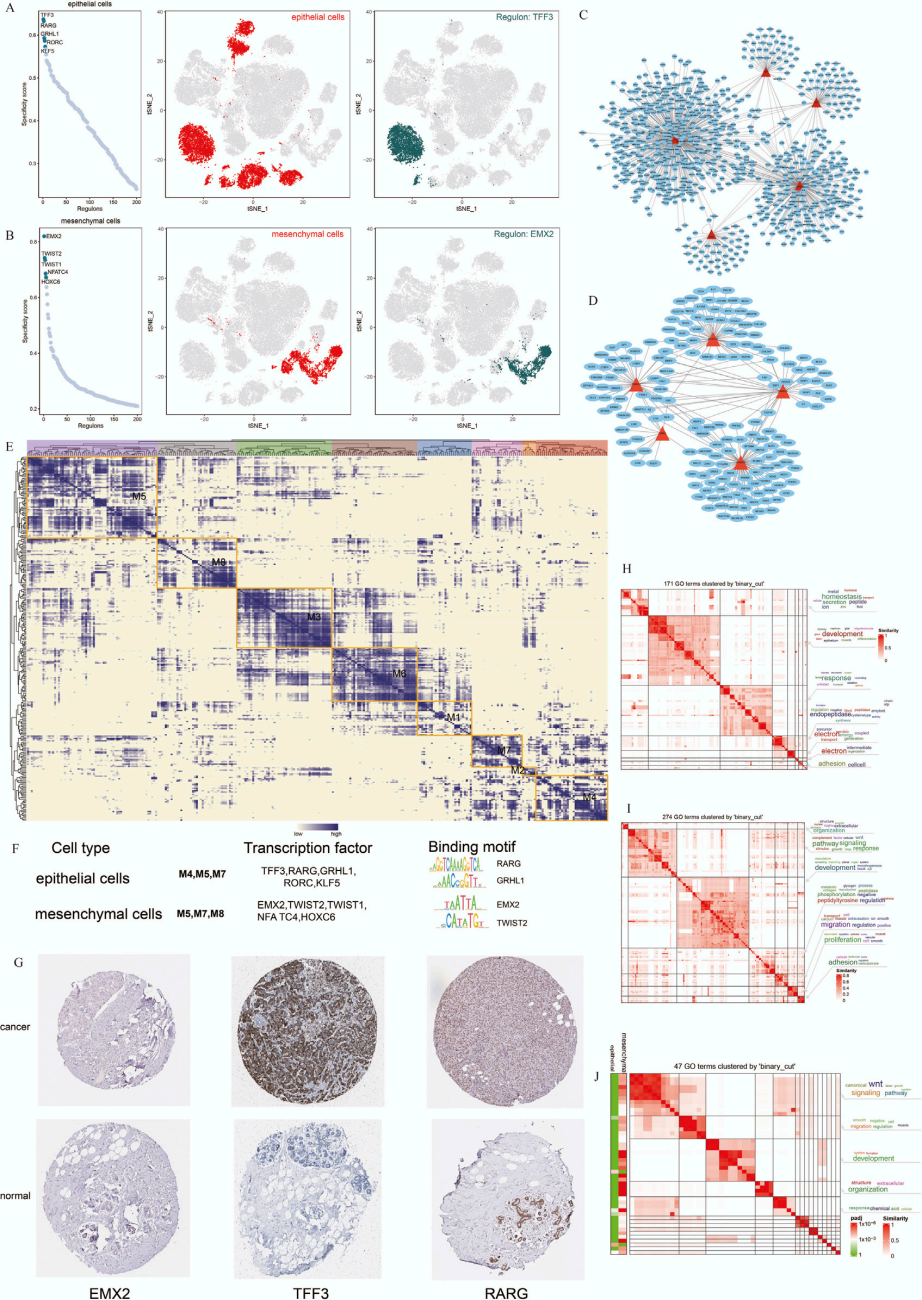
Cell Type-specific Regulon Activity Analysis. **(A,B)** Regulon rankings in epithelial and mesenchymal cells based on specificity score (RSS). **(C,D)** Top 5 TFs and transcriptional regulatory networks in epithelial and mesenchymal cells.** (E)** Regulon modules identified via the connection specificity index (CSI) matrix.**(F)** Representative TFs, binding motifs, and associated cell types for each module. **(G)** Immunohistochemistry of representative TFs in TNBC. **(H–J)** GO enrichment clustering of marker genes and similar clustering results for epithelial and mesenchymal cells.

To better understand the regulatory networks maintaining cell identity in TNBC, we used SCENIC to analyze TF activity across epithelial and mesenchymal cell populations. Our analysis identified TFF3, RARG, GRHL1, RORC, and KLF5 as key regulons in epithelial cells, while EMX2, TWIST1, TWIST2, NFATC4, and HOXC6 were the most specific regulons in mesenchymal cells (Figures 5A,B). These findings indicate that distinct regulatory programs govern epithelial and mesenchymal cell identity in TNBC.

We further constructed transcriptional regulatory networks for epithelial and mesenchymal cells to reveal how these TFs interact to maintain cell-specific gene expression profiles (Figures 5C,D). Modules containing TFF3, RARG, and GRHL1 were particularly enriched in epithelial cells, suggesting their critical role in maintaining epithelial characteristics (Figure 5E).

To validate our predictions, we used immunohistochemistry data from the Human Protein Atlas (HPA) to assess the expression of these TFs in breast cancer samples. TFF3 and RARG were strongly expressed in breast cancer tissues, supporting their involvement in TNBC pathogenesis (Figure 5G).

Additionally, we performed GO enrichment analysis to explore the biological processes regulated by these TFs in the epithelial and mesenchymal cells. Epithelial cells were predominantly associated with developmental pathways and proteolysis, whereas mesenchymal cells were enriched in signaling pathways related to migration and the WNT pathway (Figures 5H,I). Comparing these 2 cell types, we observed overlapping pathways involved in cell migration and signal transduction, suggesting that shared regulatory mechanisms contribute to cancer progression (Figure 5J).

## 4 Discussion

In this study, we generated a spatial cell atlas of TNBC using an integrative multi-omics approach that combined scRNA-seq and spatial transcriptomics. This integrative strategy overcame the limitations of individual approaches and allowed us to map the complex spatial microenvironment of TNBC. By annotating scRNA-seq data and identifying cancerous cells in spatial pathological sections, we revealed distinct spatial communication patterns among different pathological regions of TNBC. Our analysis demonstrated significant heterogeneity in cell types and gene expression across spatial regions, with normal regions primarily enriched in insulin resistance functions, whereas cancerous regions displayed a more diverse and complex cellular composition.

Our findings align with those of recent studies that have emphasized the importance of spatial heterogeneity in TNBC. For example, Shiao et al. (2024). demonstrated the value of integrating spatial and single-cell transcriptomic data to reveal the unique microenvironmental niches within breast cancer tissues, particularly highlighting the role of metabolic pathways in tumor-normal interaction. Our data showed that insulin resistance pathways were highly enriched in normal regions, suggesting a protective role against cancer progression, which is consistent with the findings of Arner and Rathmell, (2023), who linked metabolic reprogramming in the TME to immune infiltration and cancer suppression.

**FIGURE 6:**
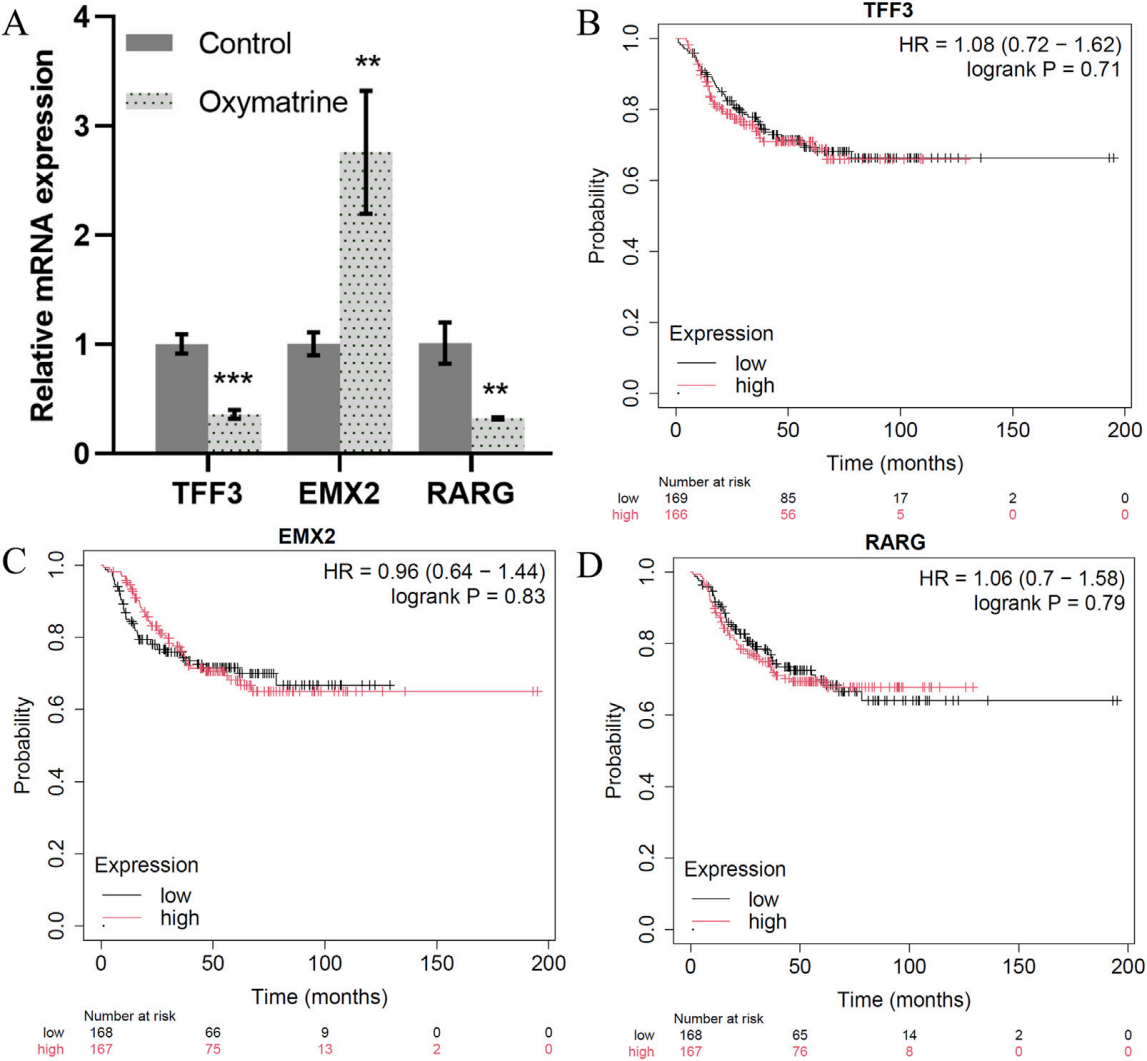
Key transcription factors. **(A)** The expression of TFF3, EMX2 and RARG mRNA as detected by qRT-PCR. **(B–D)**. Correlation between the TFF3, EMX2 and RARG gene expression in tumor and overall survival in independent cohort of TNBC patients.

Our TF network analysis further highlighted critical regulators of TNBC progression. TFF3, RARG, GRHL1, RORC, and KLF5 were identified as key regulators in epithelial cells, while EMX2, TWIST1, TWIST2, NFATC4, and HOXC6 played essential roles in mesenchymal cells and TNBC prognosis (Supplementary Material File 2). To validate the transcription factor, we treated the triple-negative breast cancer MCF7 cell line with positive drugs (oxymatrine). The expression of TFF3 and RARG decreased significantly after oxymatrine treatment but increased expression of EMX2 after treatment with oxymatrine (Figure 6A). To explore the clinical relevance of these TFs, we conducted survival analysis using KM-plotter. While TFF3 and RARG expression correlated with poorer prognosis when elevated, EMX2 displayed a non-significant trend toward favorable outcome (HR = 0.96, P > 0.7). The lack of statistical significance may be due to insufficient stratification, limited TNBC samples, and tumor heterogeneity (Figures 6B–D). TFF3 is significantly upregulated in breast cancer and is sufficient to initiate tumorigenesis. It promotes malignant transformation by activating the STAT3 signaling pathway, thereby regulating genes involved in cell cycle progression and survival, and its expression is positively correlated with advanced clinicopathological features of breast cancer (Pandey et al., 2018). RARG by signaling pathway may contribute to tumor progression by modulating cellular differentiation, proliferation, and apoptosis (Illendula et al., 2020). GRHL1, a key transcription factor involved in epithelial cell differentiation and maintenance, play a role in breast cancer progression by influencing epithelial-mesenchymal transition (EMT) and cell proliferation (Ming et al., 2018; He et al., 2021). RORC plays a crucial role in the immune system and may affect the tumor immune microenvironment of breast cancer by modulating immune cell functions (Zeng et al., 2024). KLF5 is highly expressed in triple-negative breast cancer and promotes cell proliferation, stemness, migration, and metastasis by regulating downstream target genes such as TNFAIP2 and XPO1. The stability of KLF5 is modulated by deubiquitinating enzymes BAP1 and USP3 (Qin et al., 2015; Jiang et al., 2022). These findings are consistent with the well-established role of epithelial-to-mesenchymal transition (EMT) in cancer metastasis. A recent study by Jin et al. (2021) also identified TWIST1 as central regulators of EMT, reinforcing our results that EMT-related TFs drive the mesenchymal state and contribute to the aggressive behavior of cells. EMX2 exerts tumor-suppressive functions in TNBC by directly binding to the E-cadherin promoter to maintain the epithelial phenotype and by repressing the expression of key EMT transcription factors such as TWIST1, thereby inhibiting EMT and cancer stem cell (CSC) properties (Wang et al., 2019; Zhang et al., 2023). In contrast, TWIST1 and TWIST2, as central EMT drivers of the Twist family, promote malignant progression by suppressing E-cadherin while upregulating N-cadherin and vimentin, thereby facilitating cell migration and invasion (Wang et al., 2016; Ansieau et al., 2008). NFATC4 promotes metastasis in TNBC by activating TWIST1/2 expression to synergistically drive EMT and by upregulating matrix metalloproteinases (MMP2 and MMP9) to degrade the basement membrane and enhance invasiveness (Mao et al., 2012; Kamalabadi Farahani et al., 2023). HOXC6 contributes to TNBC progression by directly binding to the promoters of TWIST1/2 to enhance their transcriptional activity and establish a HOXC6–TWIST positive feedback loop; additionally, HOXC6 activates the Wnt/β-catenin signaling pathway to maintain CSC self-renewal and engages in cross-talk with the estrogen receptor (ER) signaling pathway (Huang et al., 2022; Song et al., 2024). The shared GO functions between epithelial and mesenchymal cells, particularly in signaling pathways related to migration and development, suggest that these cells may interact and contribute to cancer dissemination through similar mechanisms.

The TME is a critical component of tumor progression and metastasis. Beyond epithelial cells, the TME comprises immune cells, fibroblasts, and endothelial cells, all of which interact with cancer cells to regulate tumor progression. While scRNA-seq has been extensively applied to investigate the TME, the mechanisms by which cancer cells influence or “domesticate” these non-epithelial cells remain insufficiently explored. Our study provides new insights into this interaction, particularly through the discovery of specific receptor-ligand pairs such as KNG1_BDKRB2 and NRG1_ERBB4, which are associated with cancer prognosis and aggressive tumor behavior, respectively. This aligns with recent work by Parambil et al. (2024), who highlighted the role of NRG1-ERBB4 in activating pro-tumorigenic pathways, including the YAP signaling pathway, which promotes tumor cell proliferation and survival. NRG1 a member of the neuregulin protein family, promotes tumor progression in TNBC by binding to the abnormally expressed ERBB4 receptor, inducing the formation of HER2–HER3/4 heterodimers and persistently activating downstream signaling pathways such as PI3K/AKT and MAPK, thereby enhancing tumor cell proliferation and resistance to apoptosis (Yun et al., 2018; Miano et al., 2022). KNG1 upon cleavage, generates bradykinin, which binds to the B2 receptor (BDKRB2) and activates inflammatory and angiogenic pathways including NF-κB and VEGF (Rex et al., 2022; Liu et al., 2017; Terzuoli et al., 2014); in the TNBC microenvironment, this signaling axis enhances vascular permeability and recruits immunosuppressive cells, thereby promoting tumor invasion and metastasis (De Visser and Joyce, 2023; Lu et al., 2025).

Interestingly, we observed that immune cells were more abundant in the normal regions of TNBC tissues, whereas cancerous regions exhibited more diverse cellular compositions. This suggests that the immune landscape in TNBC may be spatially regulated and may have important implications in immunotherapy strategies. Chen et al. (2024) recently reported that spatial differences in immune cell infiltration are critical for the effectiveness of immunotherapies in breast cancer, supporting our observations that immune cell distribution could be leveraged for targeted interventions. Our findings support the idea that oxidative stress and pathways such as TGF-β/Smad and Wnt/β-catenin may contribute to the epithelial-mesenchymal transition, as previously reported (Chen et al., 2021).

While our findings are promising, there are some limitations to this study. The integration of scRNA-seq and spatial transcriptomics relies on cellular marker genes, which can vary depending on the TME and individual cancer cases. Moreover, the spatial transcriptomic resolution remains below single-cell precision, potentially introducing inaccuracies in cell-type annotation. As spatial transcriptomic technologies advance, future studies will achieve higher resolution and provide a more comprehensive understanding of the TNBC microenvironment. Gulati et al. suggested that the latest advances in spatial resolution may soon enable near single-cell precision in spatial transcriptomics, which will further refine our understanding of tumor heterogeneity (Gulati et al., 2024).

In conclusion, our study provides a detailed spatial map of the TNBC microenvironment and identifies key transcriptional regulators involved in cancer progression. These findings identified potential therapeutic targets for TNBC and provided a foundation for investigating the spatial dynamics of cancer cell interactions and their treatment implications. With advancements in integrating spatial and single-cell transcriptomics, these technologies are expected to become critical for developing precision oncology strategies tailored to the unique spatial features of individual tumors.

## Data Availability

The original contributions presented in the study are included in the article/Supplementary Material, further inquiries can be directed to the corresponding authors.
