# Correction: Multi-omics integration uncovers key transcriptional regulators in triple-negative breast cancer spatial heterogeneity

**DOI:** 10.3389/fgene.2025.1701926

**Published:** 2025-10-07

**Authors:** Ning Zhang, Ye Zhang, Rui-Fei Yang, Min Tan, Hai-Qing Chu, Abdur Rehman, Lu-Yu Yang, Ya-Yu Li, Fahad M. Alshabrmi, Xin Zhou, Feili Xu, Shou-Ping Gong, Hui-Ling Cao

**Affiliations:** ^1^ Xi’an Key Laboratory of Basic and Translation of Cardiovascular Metabolic Disease, Xi’an Key Laboratory of Autoimmune Rheumatic Disease, College of Pharmacy, Xi’an Medical University, Xi’an, China; ^2^ College of Life Sciences, Northwest A&F University, Yangling, Shaanxi, China; ^3^ College of Health Management, Shangluo University, Shangluo, Shaanxi, China; ^4^ Xi’an Siyuan University, Xi’an, China; ^5^ Department of Medical Laboratories, College of Applied Medical Sciences, Qassim University, Buraydah, Saudi Arabia

**Keywords:** triple-negative breast cancer, spatial transcriptomics, microenvironment, tumor spread, cell communication

In the published article there was an error in the **Author List**. Authors Min Tan, Fahad M. Alshabrmi and Feili Xu were omitted as authors in the published article. The correct author list reads:

Ning Zhang^1,2,3,†^, Ye Zhang^1,†^, Rui-Fei Yang^1,†^, Min Tan^4^, Hai-Qing Chu^1^, Abdur Rehman^2^, Lu-Yu Yang^2^, Ya-Yu Li^2^, Fahad M. Alshabrmi^5^, Xin Zhou^1^, Feili Xu^3^, Shou-Ping Gong^1,*^ and Hui-Ling Cao^1,*^

1 Xi’an Key Laboratory of Basic and Translation of Cardiovascular Metabolic Disease, Xi’an Key Laboratory of Autoimmune Rheumatic Disease, College of Pharmacy, Xi’an Medical University, Xi’an, China

2 College of Life Sciences, Northwest A&F University, Yangling, Shaanxi, China

3 College of Health Management, Shangluo University, Shangluo, Shaanxi, China

4 Xi’an Siyuan University, Xi’an, China

5 Department of Medical Laboratories, College of Applied Medical Sciences, Qassim University, Buraydah, Saudi Arabia

The **Author Contributions** Statement has been corrected to read:

NZ: Data curation, Investigation, Writing – original draft, Resources, Software, Writing – review and editing.

YZ: Investigation, Resources, Writing – original draft, Data curation.

R-FY: Data curation, Writing – original draft.

MT: Writing – original draft, Writing – review and editing, Data curation.

H-QC: Writing – original draft, Validation.

AR: Writing – review and editing.

L-YY: Methodology, Software, Writing – review and editing.

Y-YL: Data curation, Writing – review and editing, Formal Analysis.

FA: Writing – review and editing.

XZ: Visualization, Writing – review and editing, Funding acquisition, Formal Analysis.

FX: Data curation, Writing – review and editing.

S-PG: Project administration, Writing – review and editing.

H-LC: Investigation, Writing – review and editing, Resources, Project administration.

The original article has been updated.

