# Supplementary figures and images for: Multi-omics integration uncovers key transcriptional regulators in triple-negative breast cancer spatial heterogeneity
Source: Front Genet. 2025 Sep 3;16:1614254. doi: 10.3389/fgene.2025.1614254 (PMC12440963; doi:10.3389/fgene.2025.1614254)

Supplementary files 2

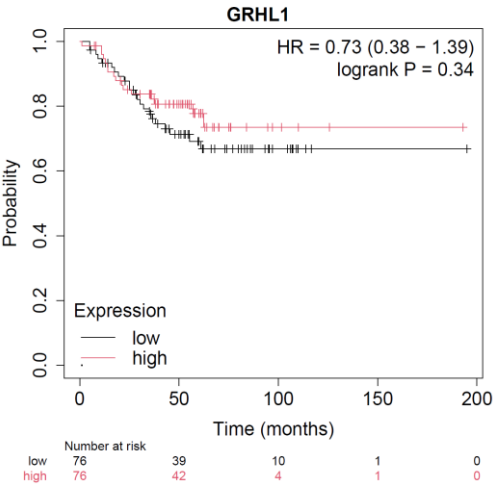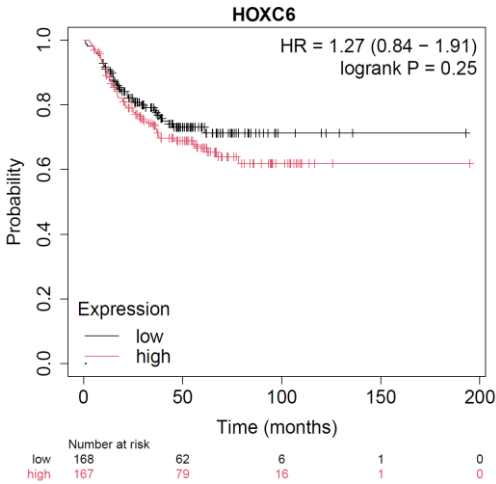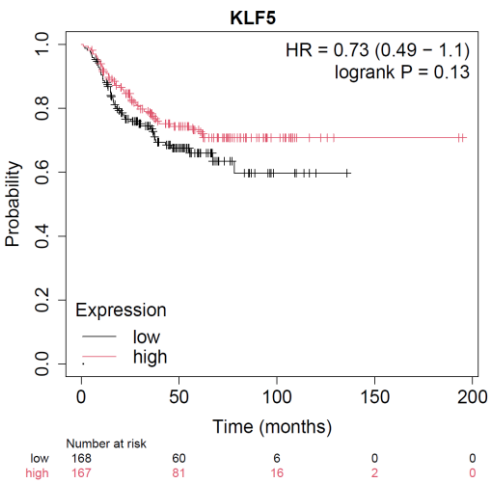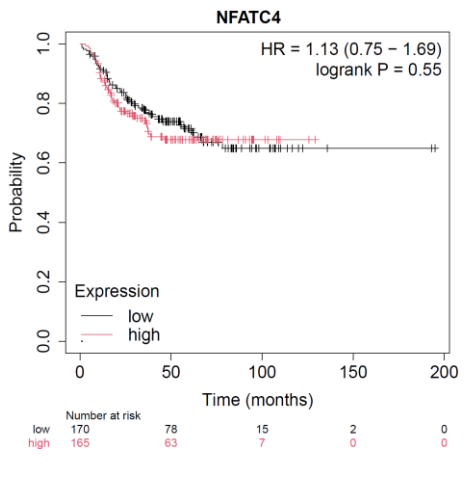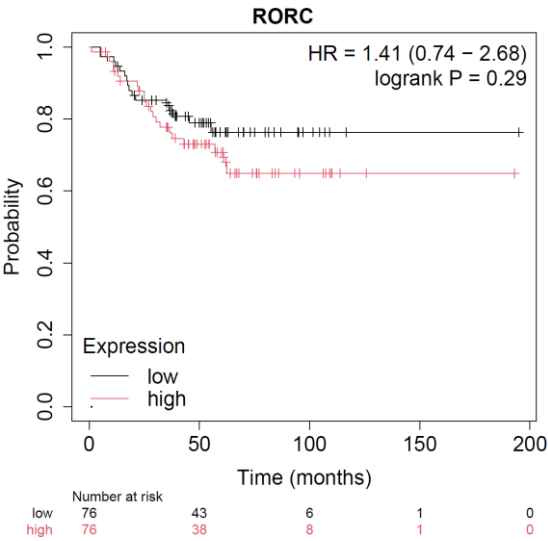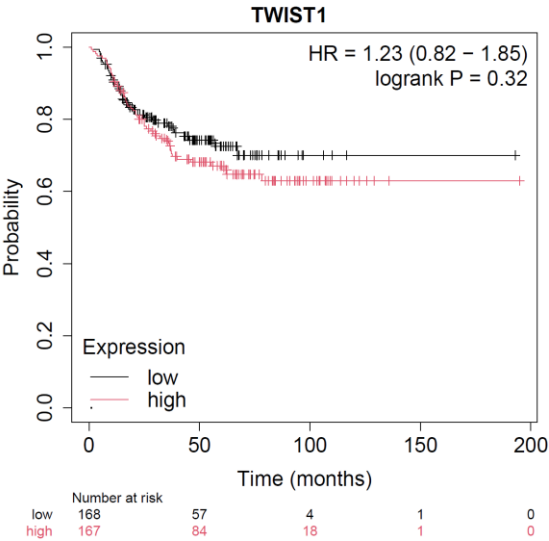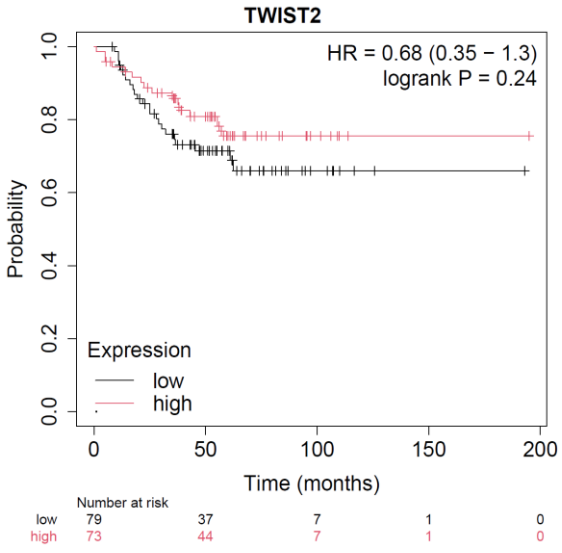

Supplement: Supplementary file 1 [file DataSheet2.pdf]
