# Supplementary material for: Multi-omics integration uncovers key transcriptional regulators in triple-negative breast cancer spatial heterogeneity
Source: Front Genet. 2025 Sep 3;16:1614254. doi: 10.3389/fgene.2025.1614254 (PMC12440963; doi:10.3389/fgene.2025.1614254)
Supplement: Supplementary file 2 [file DataSheet1.pdf]

Supplementary Table 1. Patient cohort details

Clinical and pathology details for breast cancer patients analysed by scRNA-Seq and spatial transcriptomics in this study.

data type	Case ID	Gender	Age	Grade	Cancer Type	Treatment status	Details of trea	Notable Pathological features	Stage
scRNA-Seq	44041	Female	35	3	IDC	Naïve	-	Associated high grade DCIS and focal LVI.	pT2, N1a, Mx
scRNA-Seq	44991	Female	47	3	IDC	Naïve	-	BRCA2 mutation	
scRNA-Seq	44971	Female	49	3	IDC	Naïve	-	Highly atypical cells with circumscribed periphery, associated high grade DCIS and LVI. Accompanying lymphoid stroma.	pT2, N1a, Mx
scRNA-Seq	3946	Female	52	3	IDC	Naïve	-	Basal phenotype. Reactive lymphoid infiltrate with germinal centres.	pT2, N0, Mx
scRNA-Seq	4523	Female	52	3	MBC	Treated	Neoadjuvant AC (4x), Paclitaxel (1x)	Metaplastic carcinoma with sebaceous differentiation. LVI present. RCB-II, partial pathological response to chemotherapy	pT2, pN0 (i+), pM0, Stage IIA
scRNA-Seq	4465	Female	54	3	IDC	Naïve	-	Basal phenotype - patchy CK5/6 and p63 positivity. Associated high grade DCIS at periphery of tumour mass.	PT2, N0(sn) Mx
scRNA-Seq	3963	Female	61	3	IDC	Treated	Herceptin (administered for Dx 3 years prior)	Probable recurrence from 3 years prior	pT2, pN0, Mx, Stage IIA
scRNA-Seq	4495	Female	63	3	IDC	Naïve	-	Medullary features	pT1c, pN0
scRNA-Seq	4515	Female	67	3	IDC	Naïve	-	Basal phenotype: CK5/6+ focal 40%, CK14+ focal 30%. Associated high grade DCIS and patchy lymphoid infiltrate.	PpT1c, pN1, Mi, Stage IIA
scRNA-Seq	4513	Female	73	3	MBC	Treated	Neoadjuvant AC (4x), Paclitaxel (3x)	areas of sarcomatous appearance and inflammatory infiltrate. LVI present. RCB-II, partial pathological response to	pT3, pN0, Mx, Stage IIB
Spatial Transcriptome	44971	Female	49	3	IDC	Naïve	-	Highly atypical cells with circumscribed periphery, associated high grade DCIS and LVI. Accompanying lymphoid stroma.	pT2, N1a, Mx
